# Painless retrograde type A aortic dissection followed conservative treatment of type B aortic dissection: a case report

**DOI:** 10.1186/s12872-020-01331-5

**Published:** 2020-01-13

**Authors:** Yongle Ruan, Zhiwei Wang, Zhiyong Wu, Wei Ren, Zongli Ren, Anfeng Yu, Mohamed Rahouma

**Affiliations:** 1 Department of Cardiovascular Surgery, Renmin Hospital of Wuhan University, Zhangzhidong Road, Wuhan, Hubei Province 430060 People’s Republic of China; 2 Department of Cardiothoracic Surgery, Weill Cornell Medicine, New York-Presbyterian Hospital, New York, NY 10065 USA

**Keywords:** Retrograde type a aortic dissection, Painless, Open surgical procedure

## Abstract

**Background:**

Retrograde type A aortic dissection (RTAD) is a fatal aortic disease secondary to descending aortic dissection, and might be misdiagnosed due to its atypical symptoms lead to catastrophic outcomes.

**Case presentation:**

We herein reported a case of a 40-year old Chinese non-comorbid man who received conservative treatment for acute type B aortic dissection and progressed to RTAD in a painless manner in a week. After open surgical aortic repair with stented elephant truck technique, the patient survived without obvious complication and cured with a satisfactory outcome in a half-year follow-up.

**Conclusion:**

This case indicates that RTAD may present without typical symptoms, early diagnosis and open surgical procedure are imperative for treating RTAD.

## Background

Aortic dissection is one of the most fatal acute aortic syndromes. For therapeutic purposes, aortic dissection is classified into type A and type B using the Stanford system. Type A dissection involves the ascending aorta and requires surgical management, while type B dissection does not involve the ascending aorta and requires non-surgical management in most of the cases [1]. Nowadays, thoracic endovascular aortic repair (TEVAR) has been used with increasing frequency for management of type B aortic dissection, as it is less invasive and associated with a lower complication rate compared to traditional surgical repair. Moreover, endovascular treatment of type B aortic dissections presented a lower in-hospital mortality rate (10.6%) compared to that of patients under open surgical repair (33.9%) [2]. However, RTAD can be triggered in the fragile aortic wall of acute type B aortic dissection in TEVAR procedure, and it is a fatal complication with up to 42% mortality [3]. On this basis and for risk reduction, two weeks conservative management ahead of TEVAR is recommended in acute type B aortic dissection patients [4].

Although abrupt onset thoracic pain is one of the most common symptoms in acute aortic dissection, there is still around 6% painless aortic dissection patients, and most of them are type A aortic dissection [5]. It is usually difficult to establish diagnosis and interventions in RTAD patients without typical chest pain. Herein, we present a case of a painless RTAD patient treated successfully.

## Case presentation

**Fig. 1:**
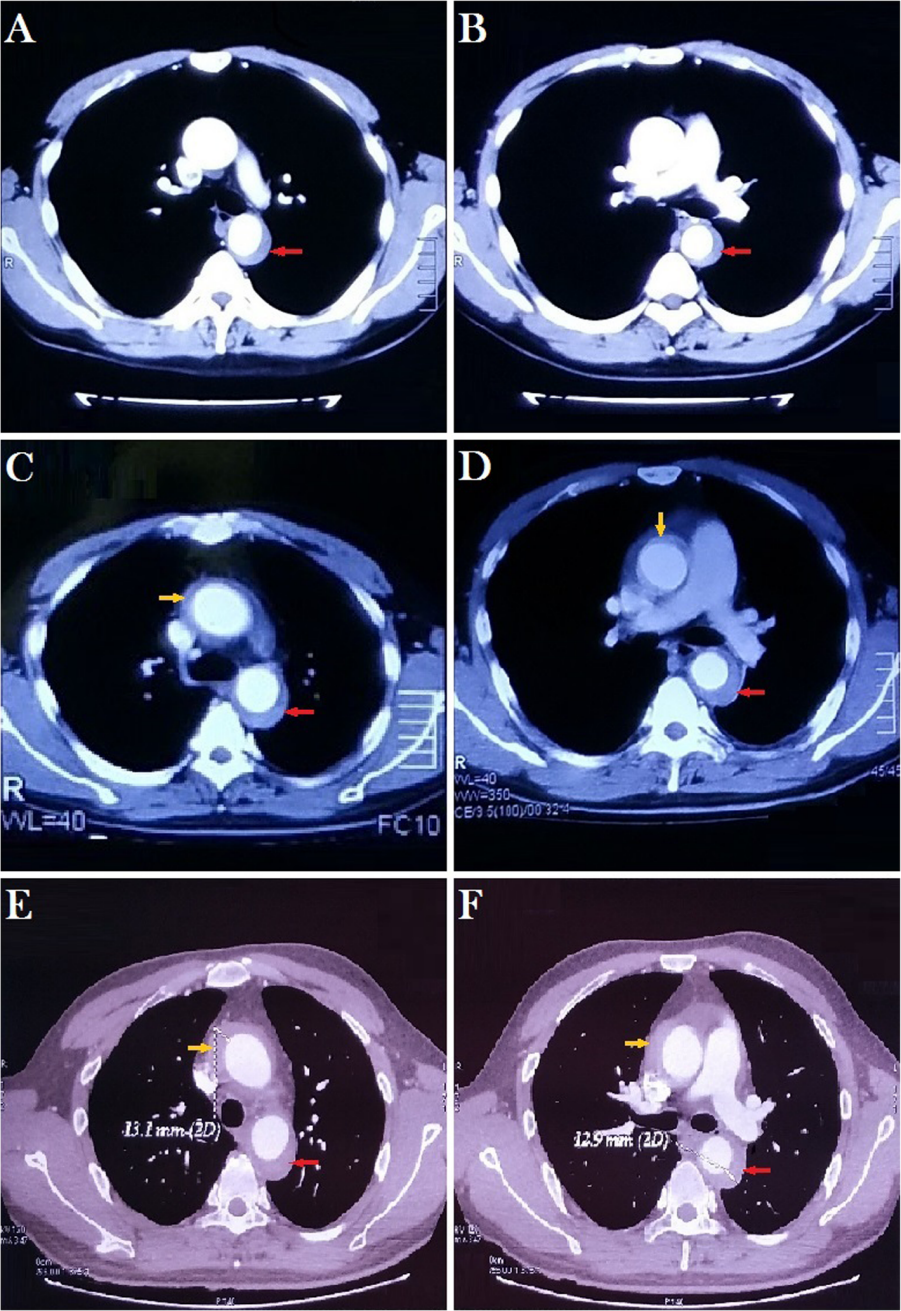
Preoperative enhanced computed tomography angiography (CTA) findings; (**A, B**): Presence of aortic dissection of the descending aorta on day 1 after disease onset (red arrow); (**C, D**): false lumen of ascending (yellow arrow) and descending aorta (red arrow) were seen on day 6; (**E, F**): Continuous thickening of false lumen in the ascending (yellow arrow) and descending aorta (red arrow) on day 12

A 40-year old Chinese male non-diabetic, non-hypertensive patient presented at our hospital with precordial and back pain that started 5 h earlier, and chest pain lasted for only a few minutes. Vital signs were recorded on admission with normal blood pressure in arms and legs. Blood pressure in the right arm was 138/74 mmHg, heart rate was 108 beats per minute and body temperature was 37.3 C degree. The patient received computed tomography angiography (CTA) and it revealed aortic dissection of the descending aorta without pericardial effusion or significant coronary disease. No dissection was found in ascending aorta, and the arterial bifurcation of the limbs and organs were not involved. The false lumen of descending aortic showed crescent-shaped with a thickness of 5 mm (Fig. 1a, b). As no poor perfusion, aneurysm, or uncontrolled pain complicated, the patient deemed not to be in need of emergency surgical repair. On this basis, conservative medical therapy was given together with blood pressure and heart rate tight control, as well as hemostasis. The blood pressure was kept less than 110/70 mmHg and the heart rate was maintained below 70 bpm after administration of calcium channel blocker (nifedipine started with 10 mg, three times a day) and beta-blocking agent (metoprolol started with 47.5 mg, once a day), the medicine dosages were adjusted according to the level of blood pressure. The patient did not experience any recurring chest pain after resolution of the original attack, which lasted a few minutes only.

**Fig. 2:**
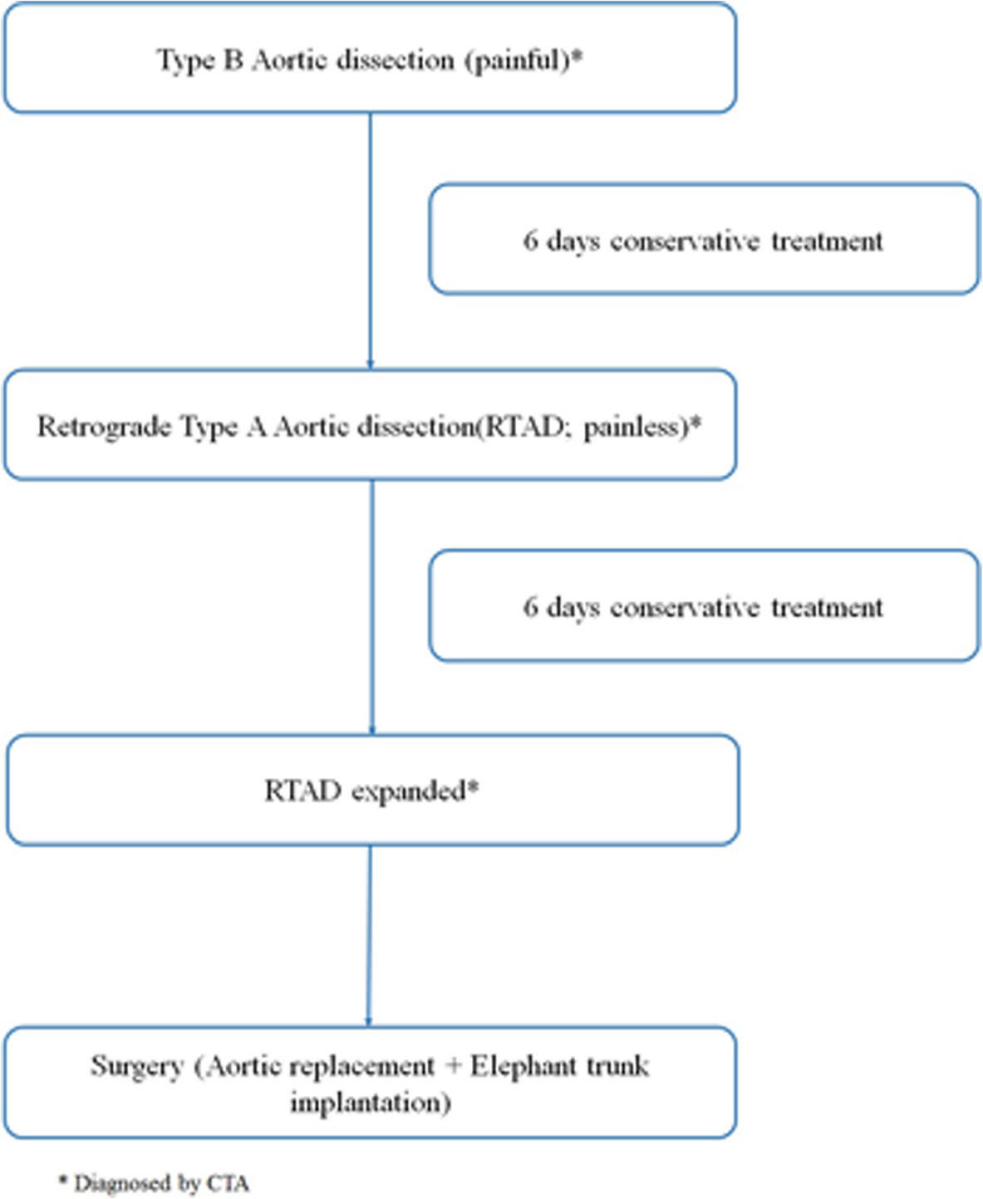
Flowchart for our case management

CTA was performed after 6 days of conservative treatment, RTAD was detected in the absence of typical thoracic pain. Ascending aortic false lumen was 5 mm thickening in a crescent shape, as well as the thickness of false lumen in the descending aorta wall was expanded to 9.5 mm (Fig. 1c, d). Subsequently, a CTA scan was followed by another 6-day medical treatment due to the patient’s refusal to open surgical therapy at the beginning. The thickness of false lumen in the walls of ascending and descending aorta was 13.1 mm and 12.9 mm respectively (Fig. 1e, f). Moreover, a cardiac ultrasound indicated pericardial effusion, together with an anechoic liquid area, 5 mm thickness behind the posterior wall of the left ventricle. It showed that type B aortic dissection progressed into RTAD without any symptom. Flowchart for management of our case was shown in Fig. 2.

On day 12, as the high risk of aortic rupture and cardiac tamponade, open surgical repair was performed under the permission of the patient. Total replacement of the aortic arch combined with elephant trunk implantation through a median sternotomy under hypothermic cardiopulmonary bypass (CPB) was proceeded as explained in earlier studies [6, 7]. Cannulation for CPB was carried out through the right femoral artery and the right atrium. Cardiac protection was done through cold-blood cardioplegia perfusion. Cerebral effective protection was done using bilateral antegrade cerebral perfusion combined with deep hypothermic circulatory arrest in aortic arch surgery. After that, the aortic arch and ascending aorta were opened and inspected closely. The primary tear of aortic dissection was observed in the descending aorta. Sleeve anastomosis was performed to suture the root of the aorta. We implanted a 100 mm long stent-graft (Shanghai MicroPort Medical Corporation, China) into the descending aorta compressed as an elephant trunk and sealed off the descending aorta intimal tear. The sutural margins were then fixed using the 4–0 Prolene suture. Besides, fenestration was performed in the stented elephant trunk to simplify the procedure of anastomosis to the left subclavian artery. Then, a four-branched prosthetic graft (Terumo Corporation, Japan) replaced the ascending aorta and aortic arch. Bilateral antegrade cerebral perfusion was discontinued after anastomosis of the innominate artery and common carotid artery. CPB was gradually returned to normal flow and started to rewarm. After the reconstruction of all aortic arch vessels, the proximal anastomosis was carried out during rewarming. The CBP time was 190 min, aortic cross-clamp time was 114 min, and double cerebral perfusion time was 35 min.

The patient was sent to the intensive care unit (ICU) for further monitoring after the operation was done, weaned from ventilator 14 h after the surgery. After mediastinal drainage tube removed on the third day, postoperative total aortic CTA was performed and revealed no aberrant changes in the position, profile or size of the artificial blood vessel and the stent. The pathological examination showed that elastic fibrous tissue hyperplasia and semi-mucoid degeneration of aortic wall, there was massive hemorrhage between the aorta walls, and the results were consistent with aortic dissection. The patient was discharged with satisfactory conditions two weeks after operation, continued to take medicine (nifedipine 30 mg per day; metoprolol 47.5 mg per day) to control blood pressure and heart rate, avoid heavy physical work, and follow-up regularly. At 6 month follow-up, no obvious abnormality was detected in the implanted prosthetic graft or stent as evident by CTA.

## Discussion and conclusions

The incidence of acute aortic dissection is about six cases per 100,000 each year. The mortality rate of acute type A dissection without treatment is about 20% in 24 h, 30% in 48 h and almost half die in a week. Fatal complications are common in medical therapy patients, such as aortic rupture, cardiac tamponade, coronary ischemia, and severe aortic regurgitation [4]. Surgical repair strategy is still the main treatment for the proximal aortic dissection while endovascular intervention is the treatment option for distal aortic dissection repair [1]. TEVAR was reported as effective management of type B aortic dissection as it was less invasive compared to the open surgical procedure with higher mortality. However, the aortic wall and intimal flap are usually in a state of acute inflammation at the early stage of type B aortic dissection, and a new dissection or rupture may occur. An incidence of RTAD was reported up to 13.8% in acute type B aortic dissection patients after TEVAR, and the rate of mortality exceeded 42% [8]. Shu et al. [4] proposed a 2-week conservative therapy for type B aortic dissection ahead of TEVAR procedure, which could reduce postoperative complications effectively. Furthermore, some studies had found favorable outcomes with medical treatment in a clinically stable patient in whom the false lumen of the ascending aorta was completely thrombosed [9].

Conservative therapy as the initial approach makes sense for patients in the acute stage of uncomplicated type B aortic dissection, however, the possibility of RTAD still exists and might be overlooked. In the current case, the patient showed a painless RTAD following conservative treatment in a week, and surgical treatment was mandatory to avoid lethal consequences. Acute aortic dissection usually presents with one or more concomitant symptoms/signs such as chest pain, congestive heart failure, myocardial infarction, limbs and bowel ischemia, stroke, or paraplegia. Sudden-onset severe chest or back pain remains the most common symptom of aortic dissection. However, about 6.4% of aortic dissections are painless and most of the patients suffer from type A aortic dissection, which is associated with increased mortality [10]. As RTAD is an extreme emergency and fatal complication, rapid diagnosis and intervention are imperative. Painless RTAD must be under high clinical suspicion in acute type B aortic dissection patients involving either TEVAR or conservative management, even without the presence of a characteristic pain as supported. Unfortunately, it is more likely that there is even a higher rate of painless RTAD occurrence because of misdiagnosis and underestimation.

This case initiated with typical pain in a descending aortic dissection, but it progressed to RTAD in a painless manner. The mechanism of painless of aortic dissection remains incompletely clear [11]. The innervated layer of the aortic artery is responsible for pain sensation, and the absence of chest pain may be attributed to the damage of innervation to the aorta resulting from the descending aortic dissection. Ischemic necrosis of nerves results from direct vascular compromise and can lead to specific classes of neurologic dysfunction [12]. Atypical symptoms of aortic dissection make accurate diagnosis difficult, especially in painless aortic dissections. Diagnosis of painless RTAD requires a high degree of consideration and suspicion in patients with descending aortic dissection or aneurysm. Computed tomography has high sensitivity and specificity for diagnosing aortic dissection, and CTA is a reliable way to confirm the diagnosis of painless RTAD. Open surgical repair appears optimal for repairing RTAD in the ascending aorta. As the ascending aorta was involved by dissection in this case, the surgical replacement had been proposed. Considering the technical difficulty of dealing with descending aortic lesions through a sternotomy, we selected the elephant trunk implantation to repair descending aortic lesions and to block the rupture.

Controversy remains on the optimal management choice for type B aortic dissection patients during the acute stage. As a high mortality rate, painless RTAD should be considered and diagnosed promptly in acute type B aortic dissection under conservative treatment. CTA is a reliable tool for the diagnosis of RTAD, and open surgical procedure is efficacious in treating.

## Data Availability

The datasets used and/or analyzed during the current study are available from the corresponding author on reasonable request.

## Abbreviations

CPB: Cardiopulmonary bypass
CTA: Computed tomography angiography
RTAD: Retrograde type a aortic dissection
TEVAR: Thoracic endovascular aortic repair
